# Application of *In Silico* and HTS Approaches to Identify Nuclear Import Inhibitors for Venezuelan Equine Encephalitis Virus Capsid Protein: A Case Study

**DOI:** 10.3389/fchem.2020.573121

**Published:** 2020-12-23

**Authors:** Sharon Shechter, David R. Thomas, David A. Jans

**Affiliations:** ^1^Shechter Computational Solutions, Andover, MA, United States; ^2^Department of Chemistry, College of Sciences, University of Massachusetts Lowell, Lowell, MA, United States; ^3^Nuclear Signalling Laboratory, Department of Biochemistry and Molecular Biology, Biomedical Discovery Institute, Monash University, Monash, VIC, Australia

**Keywords:** highthroughput screening, *in silico* screening, Venezuelan equine encephalitis virus (VEEV), antiviral agents, computational modeling

## Abstract

The development of new drugs is costly and time-consuming, with estimates of over $US1 billion and 15 years for a product to reach the market. As understanding of the molecular basis of disease improves, various approaches have been used to target specific molecular interactions in the search for effective drugs. These include high-throughput screening (HTS) for novel drug identification and computer-aided drug design (CADD) to assess the properties of putative drugs before experimental work begins. We have applied conventional HTS and CADD approaches to the problem of identifying antiviral compounds to limit infection by Venezuelan equine encephalitis virus (VEEV). Nuclear targeting of the VEEV capsid (CP) protein through interaction with the host nuclear import machinery has been shown to be essential for viral pathogenicity, with viruses incapable of this interaction being greatly attenuated. Our previous conventional HTS and *in silico* structure-based drug design (SBDD) screens were successful in identifying novel inhibitors of CP interaction with the host nuclear import machinery, thus providing a unique opportunity to assess the relative value of the two screening approaches directly. This focused review compares and contrasts the two screening approaches, together with the properties of the inhibitors identified, as a case study for parallel use of the two approaches to identify antivirals. The utility of SBDD screens, especially when used in parallel with traditional HTS, in identifying agents of interest to target the host–pathogen interface is highlighted.

## Introduction

Identifying and developing new drugs is notoriously difficult and expensive. Estimates from 2003 put the out-of-pocket cost of developing a drug to market approval at $US403 million, including the costs of abandoned compounds (DiMasi et al., 2003), with more recent estimations indicating inflation in the costs to almost $US1.4 billion per approved new compound (DiMasi et al., 2016), which is consistent with other studies (Morgan et al., 2011). Almost 95% of drugs entering human trials fail (Seyhan, 2019). Even in advanced phase three trials, around 50% fail during clinical development, largely because of problems of efficacy and safety (Hwang et al., 2016); despite varying approaches to drug discovery, the dropout rate remains exceedingly high.

Modern high-throughput screening (HTS) approaches are able to screen large numbers of compounds (An and Tolliday, 2010; Du et al., 2016), but require extensive investment of time and money. An important advance has been in the use of *in silico* approaches to aid drug discovery, with computational methods commonly used to filter the field of potential candidates to screen based on physicochemical properties and to identify compounds similar to active leads. Ultimately, however, experimental assessment of putative drugs is still required to identify and follow a lead. More recently, a new application of computational biology–computer-aided drug design (CADD)—has emerged to further improve the success rate of drug screening. It works as a collaborative effort between structural biologists, biophysicists, and computational scientists and is aimed at discovering new chemical entities using *in silico* modeling. CADD can reduce screening costs, help model details of drug–receptor interactions, and accelerate drug discovery and development (Mpamhanga et al., 2006; Dutta et al., 2010; Zhong and Zhou, 2014).

CADD methods can be broadly classified as either ligand- or structure-based drug design (LBDD and SBDD, respectively), depending on the availability of the target structure (Sliwoski et al., 2014; Yu and MacKerell, 2017). SBDD is based on the premise that knowledge of a receptor structure can help to rationalize and optimize the design of an active drug against it, since ligand–receptor interactions are mediated by their complementarity. LBDD, on the other hand, can be used when the three-dimensional structure of a ligand, but not that of the target receptor, is known. This ligand can be used as a template to develop a pharmacophore model to identify molecules that possess all necessary structural and chemical features to bind to the target's active site (Singh and Surabhi, 2018).

This focused review compares and contrasts conventional and *in silico* HTS approaches to identify agents targeting a specific host–pathogen protein–protein interface as a case study. It examines the nature of the compounds identified, highlighting the utility of SBDD screens, especially in terms of the new knowledge gained and in combination with traditional HTS.

## Venezuelan Equine Encephalitis Virus: A Target for Antivirals

Venezuelan equine encephalitis virus (VEEV) is a single-stranded RNA virus of the genus *Alphavirus* (Weaver and Barrett, 2004; Weaver et al., 2004). VEEV is a mosquito-borne virus that normally infects small rodents. However, mutations can enable infection of human and equine populations, notably leading to outbreaks with tens of thousands of human cases (Quiroz et al., 2009; Forrester et al., 2017). The high infectivity of VEEV and the presence of circulating virus in animal reservoirs creates a constant risk of a new outbreak (Weaver and Barrett, 2004; Weaver et al., 2004). Treatment options are limited and there is no vaccine approved for the general public (Sharma and Knollmann-Ritschel, 2019), making the development of anti-VEEV agents a high priority (Reichert et al., 2009; Chung et al., 2014; Lundberg et al., 2016; Urakova et al., 2018; Carey et al., 2019; DeBono et al., 2019).

Like other alphaviruses, one of the ways that VEEV evades the host immune response is by inhibiting host cell transcription (Fros and Pijlman, 2016). While the exact mechanism behind this is still unclear, it is known to require interactions between the VEEV capsid (CP) protein and members of the host importin (Imp) superfamily of transporters, which mediate signal-dependent trafficking into and out of the nucleus; this is essential for many key cellular processes, including the innate immune response to combat infection. VEEV CP interacts with the IMP family members exportin 1/CRM1 and a heterodimer of Impα and Impβ1 (Impα/β1) (Garmashova et al., 2007; Atasheva et al., 2008, 2010a,b; Atasheva et al., 2015). Small molecule inhibitors of either Impα/β1 (e.g., ivermectin) or CRM1 (e.g., leptomycin B) can reduce VEEV production in cell culture (Lundberg et al., 2013, 2016), but have the potential to be toxic since Impα/β1 and CRM1 are so critical to normal cell function.

**Figure 1:**
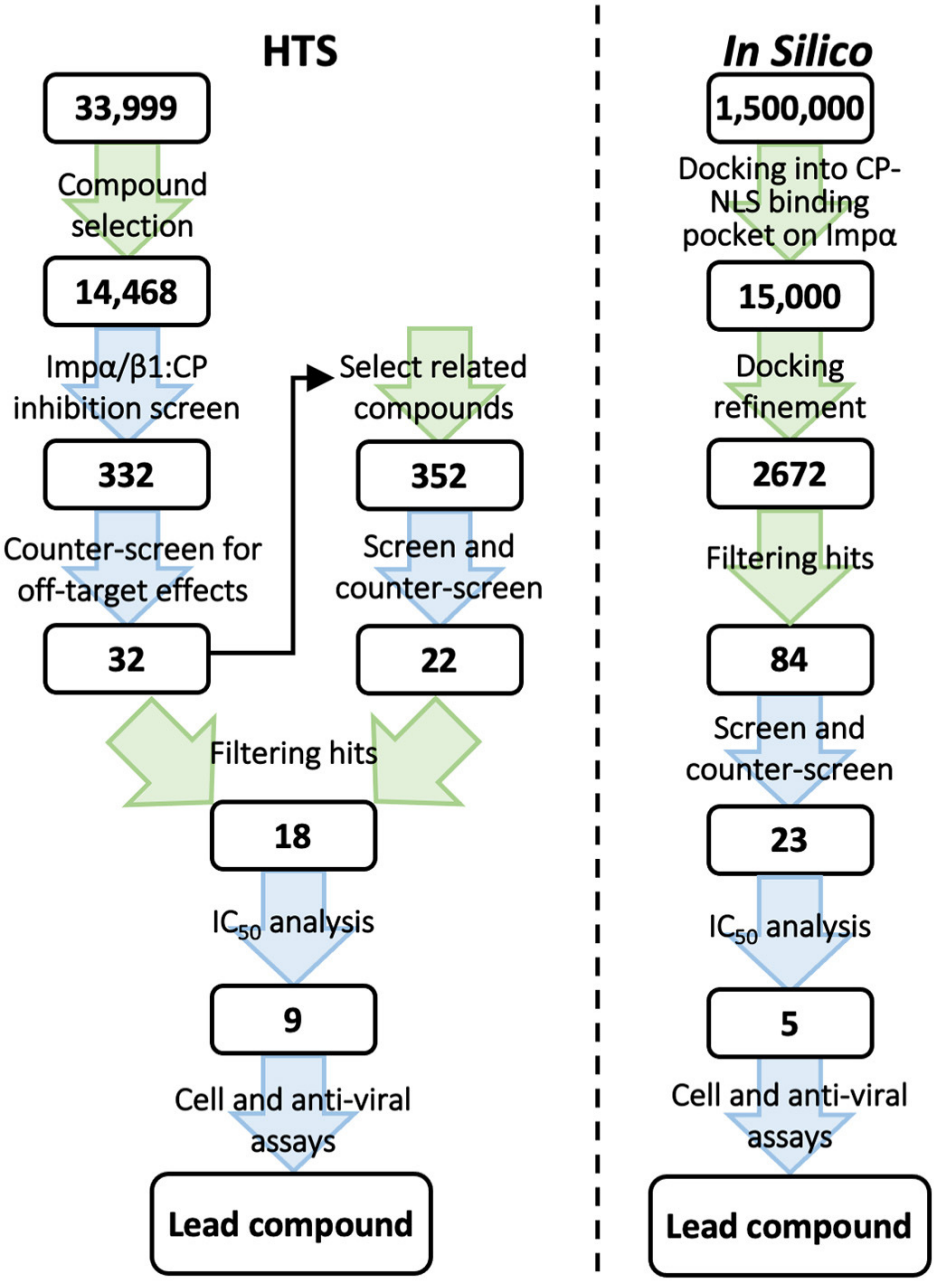
Schematic representation of the steps in our conventional HTS (left) and *in silico* screen (right) for inhibitors of the Impα/β1-CP interaction. The number of compounds under consideration at each step is indicated in the boxes. Green arrows indicate computational steps; blue arrows indicate experimental steps. IC_50_, concentration resulting in half-maximal inhibition; NLS, nuclear localization signal.

Our HTS studies set out to identify novel small molecule inhibitors that are specific for the Impα/β1–VEEV CP interaction through conventional HTS using a library of >14,000 chemical compounds (Thomas et al., 2018) in parallel with an *in silico* screen of a library of 1.5 million compounds using molecular docking approaches (Shechter et al., 2017) (Figure 1). Post screening, binding inhibition of candidate compounds was tested in an *in vitro* protein–protein binding assay using recombinantly expressed CP, Impα, and Impβ1. While both screening approaches identified novel compounds with half-maximal effective concentration (EC_50_) values of around 10 μM for antiviral activity in cell culture, there were marked differences between the properties of the lead compounds and the information gained from the two screens.

## Results of Conventional High-Throughput Screening

The compounds screened were from the Queensland Compound Library (QCL) Open Scaffolds Collection (OSC). The QCL provides access to a collection of small molecules to academic and not-for-profit organizations at reduced rates, providing an excellent starting point for our screen (Simpson and Poulsen, 2014). We first used computational approaches to filter the compounds for a variety of traits to identify more “ideal” drug candidates, including compound size, ring structure, chirality, and numbers of hydrogen bond donors and acceptors. This reduced the approximately 34,000 compounds available in the OSC to 19,408. Compounds whose structures were >90% similar to others in the set were then also eliminated, leaving a total of 14,468 compounds for the HTS itself (Thomas et al., 2018). A roboticized screening platform was used.

The HTS involved the parallel assessment of each of the compounds for the ability to reduce signal in the AlphaScreen system generated by:

1. the Impα/β1–VEEV CP target interaction
2. interaction of Impα/β1 binding with another viral protein (simian virus SV40 large tumor antigen; T-ag) (counter-screen to identify agents targeting Impα/β1 specifically, rather than the Impα/β1–VEEV CP interface)
3. a positive control reaction to identify/triage non-specific agents interfering with the AlphaScreen chemistry.

By analyzing the results for each compound for (i)–(iii), we were able to identify compounds capable of inhibiting the Impα/β1–VEEV CP target interaction selectively.

Based on the initial screen, we selected another 352 compounds that structurally resemble the best hit compounds from the HTS of the original library. These were also tested for (i)–(iii) above, resulting in a total of 54 compounds with selective activity (Figure 1, left). Eighteen of the most active compounds were assessed in detail by IC_50_ analysis (all <50 μM), with two compounds shown to reduce VEEV replication in infected cells (EC_50_ values of 11 and 27 μM) with no detectable toxicity. A third active compound was discarded because of toxicity. We also confirmed that the compounds were able to inhibit Impα/β1-dependent nuclear import of CP but not that of T-ag.

We were able to perform preliminary structure–activity relationship (SAR) analysis on the most active compound, G281-1564, as a member of a group of structurally similar compounds, for toxicity and activity. A subsequent study examining VEEV CP-induced cell cycle delay showed that G281-1564 was able to inhibit the change in cell cycle progression, but also that it induced some changes in the cell cycle on its own, likely because of off-target effects (Lundberg et al., 2018).

## Results of *In Silico* High-Throughput Screening

Our SBDD screen was based on the available crystal structure of the VEEV CP bound to the binding pocket of mouse Impα2 lacking the autoinhibitory Impβ1 binding domain (Fan, 2012; Shechter et al., 2017). The crystal structure was based on a 12-amino-acid peptide containing the VEEV CP nuclear localization signal (NLS) recognized by Impα. However, the classical Impα binding NLS is only a short K–K/R–X–K/R sequence (Marfori et al., 2012; Smith et al., 2018). Docking simulations of truncated versions of the VEEV CP peptide to Impα identified the minimal region required to model Impα binding as the core of the NLS (KKPK; amino acids 6–9) using estimates of the free energy of binding. Three-dimensional modeling of the core KKPK domain aligned well with the conformation of the CP peptide in the published crystal structure, while the non-core residues showed significant variation in their orientation. Further computational analysis confirmed these four residues as critical for Impα-CP binding, as their omission resulted in significant increases in free binding energies, theorized to correlate with decreasing binding affinities (Shechter et al., 2017). In identifying the minimal NLS sequence that would still bind strongly to Impα, we identified the key ligand–receptor interactions in the Impα-CP binding pocket that should be targeted by potential inhibitors. From the modeling of the KKPK NLS and *in silico* alanine scanning of residues located in the binding pocket, the key interacting residues in the Impα binding pocket were identified. This was then used as the basis to screen for inhibitors that can mimic the same key interactions.

**Figure 2:**
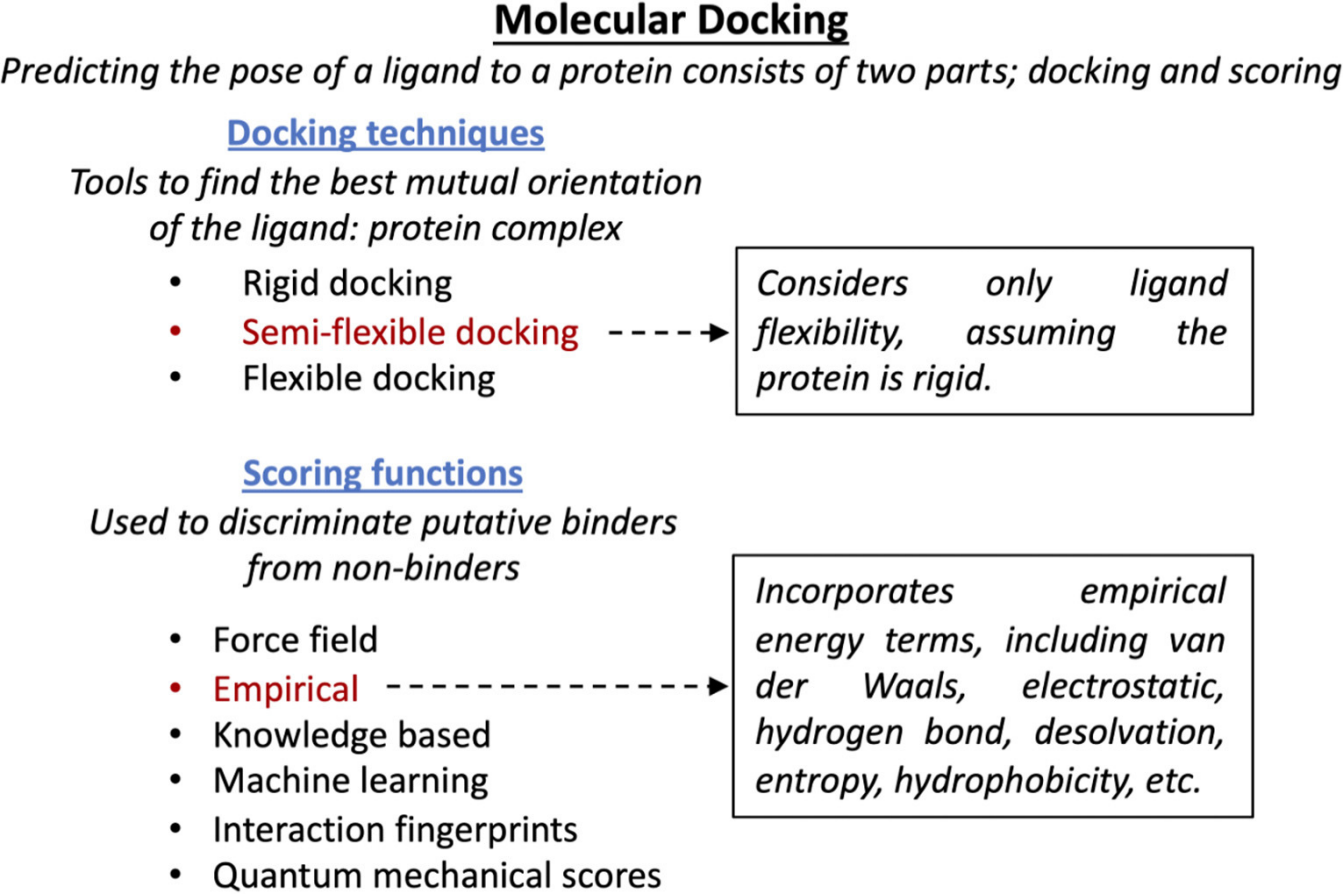
SBDD molecule docking approach. Schematic representation of computational strategies used to screen compounds binding to Impα. Semi-flexible docking provides a good compromise between accuracy and computational efficiency (Halperin et al., 2002). The empirical scoring function relies on the sum of various quantifiable interactions (Huang et al., 2010), while quantum mechanical scores are based on exact modeling and so are computationally limiting (Cavasotto and Aucar, 2020). Other approaches rely on approximations of potential energies (force field) (Brooijmans and Kuntz, 2003) or incorporating existing docking data (i.e., knowledge based, machine learning, and interaction fingerprints) (Mpamhanga et al., 2006; Khamis et al., 2015).

For the *in silico* screen, 1.5 million publicly available compounds were curated and prepared using Ligprep (Schrodinger, Portland, OR, USA) (Figure 1, right). Compounds were then docked into the CP core NLS binding pocket on Impα using semi-flexible docking (Salmaso and Moro, 2018), and scored using the Glide (Schrodinger) empirical functions (Friesner et al., 2004; Tubert-Brohman et al., 2013) (Figure 2), based on the free energy of the binding process. The top 1% scoring compounds were then assessed for occupancy of the binding site, identification of spatial clashes, and alignment with the CP NLS (Figure 2). From this, 2,672 promising hit compounds were identified. As with the HTS, removal of compounds that were structurally similar left 135 unique compounds, of which 84 could be procured for testing using the AlphaScreen binding assays used in the HTS (Figure 1, right). At 10 μM, 23 out of the 84 compounds (27%) inhibited Impα/β1-CP binding by >30%. These active compounds were then counter-screened for their ability to inhibit Impα/β1–T-ag binding. Although both CP and T-ag NLSs are believed to bind the same binding pocket on Impα, 17 of the 23 compounds were more than twice as active in inhibiting Impα/β1-CP binding as they were in inhibiting Impα/β1–T-ag binding (Shechter et al., 2017). The IC_50_ values of the most active compounds were comparable to those found in the HTS, ranging from 5 to 40 μM.

Two of the top four compounds proved to be selective for the Impα/β1-CP interaction, while the other two showed comparable activity for the Impα/β1-CP and Impα/β1–T-ag binding. In cell-based assays, the two selective compounds inhibited the nuclear accumulation of CP but not T-ag. In infectious assays, only one of the top four non-specific compounds was able to reduce VEEV replication (EC_50_ of 10 μM), although with some toxicity (CC_50_, concentration yielding 50% cytotoxicity, of 36 μM).

## Approaches to Enhance Structure-Based Drug Design

It should be noted that, based on the three-dimensional structure of the target alone as a starting point, SBDD cannot be expected to identify only selective, high-affinity compounds with favorable pharmacokinetic and pharmacodynamic properties. Rather, SBDD encompasses a range of different computational tools that can be applied systematically in different ways; for example, to provide insight into target–ligand interactions [e.g., by molecular dynamics (MD)] or to complement/enhance conventional HTS approaches through initial curation/filtering (e.g., to select active moieties or remove promiscuous compounds) to focus the search toward compounds more likely to bind the target itself. While our *in silico* screen was performed with modest computing power, additional approaches requiring more extensive resources can also be employed. This includes the use of MD simulations to account for protein flexibility, to provide detailed molecular-/atomic-level information, and to identify potential cryptic binding pockets (Nichols et al., 2011; Ferreira et al., 2015). By incorporating how interactions between a compound and the binding pocket can induce conformational changes in both the compound and binding pocket, novel scaffolds may be identified, and docking of previously identified compounds can be refined (Alonso et al., 2006; Rastelli et al., 2009; Sabbadin et al., 2014). Additionally, a secondary *in silico* screen incorporating information from active/inactive inhibitors as a guide could be performed to strengthen the binding hypothesis.

High computational power is required to screen millions of compounds in the shortest possible time, while considering/incorporating additional screening parameters/additional structures of the same targets, with the aim of enriching the sample set, toward achieving more biologically relevant “hits.” This is especially true now that the incorporation of MD simulations has become a routine part of most *in silico* screens. Although not usually available for most researchers, sufficient computational power can be achieved in various ways; for example, through consortia or collaborations and through utilizing the collective “power” of grid and distributed computing networks (Richards, 2002), or cloud computing, to enable target- and ligand-based screening of huge databases to be accomplished in the shortest possible time.

## Comparison of Outcomes From the High-Throughput Screening Approaches

**Figure 3:**
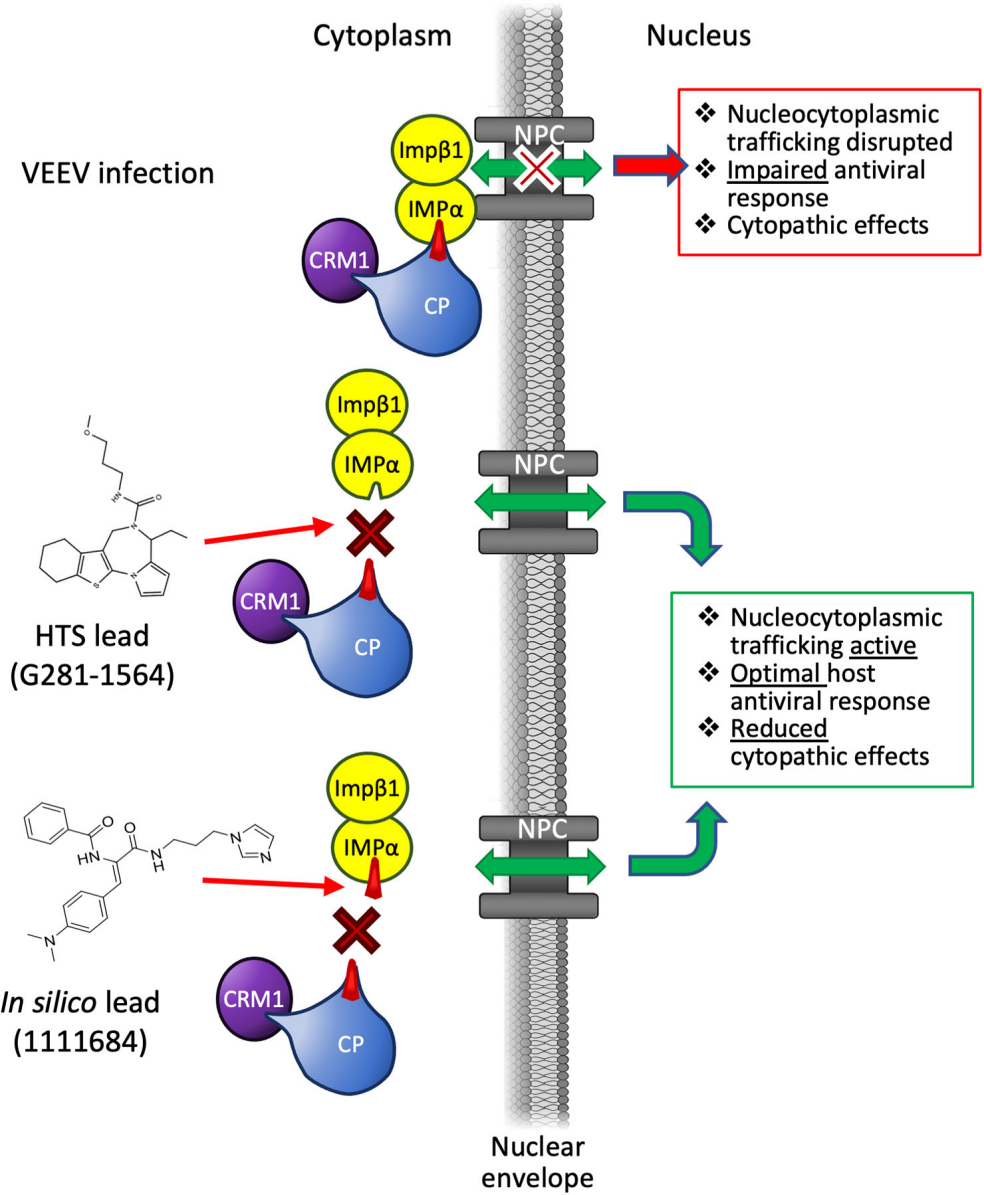
Mechanism of action of inhibitors of Impα/β1–VEEV CP Interaction. During infection, VEEV CP simultaneously binds Impα/β1 and CRM1, accumulating at the nuclear pore complex (NPC) to prevent nucleocytoplasmic transport through the NPC. This impairs the ability of the host cell to develop an antiviral response, and leads to the development of cytopathic effects. Lead compounds inhibiting CP recognition by Impα/β1 through blocking the Impα-binding pocket (1111684) or preventing binding generally (G281-1564) abrogate nuclear transport disruption, enabling an optimal antiviral response to reduce viral replication/cytopathic effects.

Although the two approaches successfully identified compounds that were able to inhibit the same target (Impα/β1-CP binding), the results were appreciably different (Table 1 and Figure 3). From the conventional HTS, two active compounds with low toxicity were identified (Thomas et al., 2018), enabling limited SAR analysis on the lead compound (selectivity index *c*.10) based on the availability of related compounds for experimental verification. However, since *in silico* docking into the Impα binding pocket was unsuccessful, further study is required to establish exactly how these compounds may interact with/perturb the Impα/β1-CP interface.

**Table 1:** Comparison of lead compounds identified through conventional or *in silico* HTS.

	**HTS lead compound G281-1564**	***In silico* lead compound** **1111684**
Structure	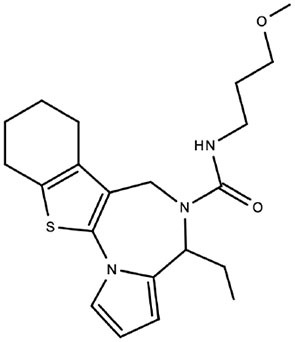	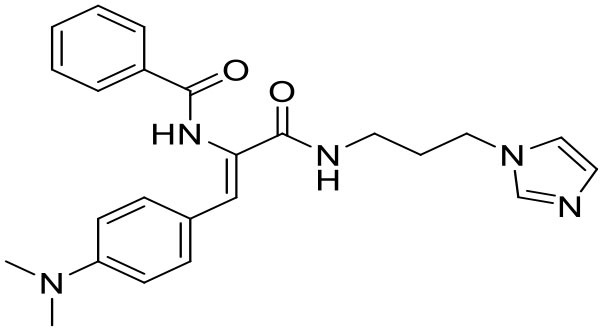
EC_50_ (μM)^a^	10.8	9.9
IC_50_ (μM)^b^	25	5.2
CC_50_ (μM)^c^	>100	36.4
Notes	• Preliminary SAR produced • Unknown binding target • Specifically inhibits Impα-CP binding	• No SAR available • Binding pose modeled • Non-specifically inhibits Impα binding

a *Concentration at which there is 50% of maximal inhibition of viral replication during infection of Vero cells*.

b *Concentration at which there is 50% of maximal inhibition of Impα/β1-CP binding in an AlphaScreen assay*.

c *Concentration at which there is 50% of maximal toxicity in Vero cells*.

*SAR, structure–activity relationship*.

By comparison, the *in silico* screen identified only one antiviral compound, which possessed some toxicity (selectivity index of *c*.4) (Shechter et al., 2017). The compound was also large and overly flexible in structure to be easily manipulated for SAR/medicinal chemistry optimization, making it of limited usefulness as a candidate for drug development. However, as a screen for inhibitors of Impα/β1-CP binding, it identified a small list of putative hits, of which 27% were confirmed to be active (a 75-fold enrichment of hits compared with our conventional HTS). Importantly, the compounds that inhibited binding *in vitro* provided valuable information on Impα/β1–NLS ligand binding, with *in silico* modeling identifying key interactions of the compounds with Impα, and in particular identifying residues important for general ligand binding and those more specific to CP interactions. A major outcome of the *in silico* analysis was the demonstration that compounds targeting the NLS binding pocket on Impα could be designed that were able to inhibit specific ligands and not others, to reduce off-target effects/potential toxicity. Clearly, specifically targeting the NLS-binding pocket of Impα to generate antiviral compounds has enormous potential for the future.

At face value, our conventional HTS provided more candidate hit compounds with demonstrable antiviral activity than the *in silico* screen; that there was no limit on the domain targeted (unlike the *in silico* screen) enabled a greater range of compounds to be identified. However, this is also consistent with the greater potential for identified compounds to possess off-target effects (see Lundberg et al., 2018), which is, of course, an important consideration for future drug development. The ability of HTS to identify compounds with unexpected mechanisms cannot be replicated *in silico* at present, meaning that HTS will likely remain a key approach in drug discovery. As indicated, an advantage of the *in silico* screen is the information provided on the binding of compounds and ligands to the Impα-NLS binding pocket. Using this information in parallel with testing the compounds identified in the screen for their ability to inhibit other proteins recognized by Impα, a detailed map of interactions in the Impα binding pocket could be established, which would be of great value to researchers in the future.

## Alternative *In Silico* Approaches

Our *in silico* approach was intended to screen existing libraries of molecules to facilitate the transition from screening hit to testing for antiviral activity, as any lead compounds would be readily obtainable for experimentation, and with varying degrees of information already available for them. Alternative *in silico* approaches include LBDD (see above), which could be applied to find mimics of the VEEV CP NLS pharmacophore. SAR analysis of the activities of a range of compounds would provide a starting point to enable additional potentially active compounds to be identified, whereas inactive compounds could be used to eliminate unwanted interactions or volumes in the binding pocket that should not be used. From the initial dataset, active compounds could be predicted through LBDD pharmacophore modeling, similarity searching (Yang, 2010; Yu and MacKerell, 2017), or quantitative SAR (QSAR) analysis. QSAR calculates a range of descriptors such as physicochemical, electronic, topological, and shape properties. Lipinski's rule of five (Lipinski et al., 2001) is a classic example of a straightforward application of QSAR where bioavailability is related to descriptors including the octanol–water partition coefficient (logP), molecular weight, number of hydrogen bond donors and acceptors, and number of rotatable bonds. In contrast, LBDD pharmacophore modeling superposes a set of active molecules and extracts common chemical features that are essential for their bioactivity. In general, pharmacophore generation from multiple ligands involves two main steps: handling the conformational flexibility of ligands and conducting molecular alignment. “Similarity searching” (Yu and MacKerell, 2017) measures and ranks library compounds for similarity to active reference compounds or compounds that possess desired properties, based on the assumption that similar structures have similar properties in terms of activities/mechanism/target; multiple rounds of experimental assessment and *in silico* analysis are usually required to develop a relevant model to identify active compounds and avoid non-specific inhibitors.

Fragment-based docking enables new custom compounds to be designed/built, as opposed to repurposing existing ones, and can be performed *in silico* (Anderson, 2003) or experimentally (Erlanson, 2012). This approach aims to generate novel compounds by docking small molecule fragments to the binding site, scoring them, and growing active fragments into drug-like compounds by linking with other docked fragments. It enables molecules and docking modes to be investigated that can fully exploit possible binding site(s)/binding modes to enable significantly more structural permutations to be considered/tested. Difficulties with this approach, however, include the issue of how to identify suitable fragments that can be predicted to bind selectively to the site of interest, and how to optimize merging of these fragments into a functional molecule without distorting individual binding modalities. Finally, the ensuing medicinal chemistry challenge of synthesizing the merged compounds gives no guarantee that the end-product will possess the expected functionality. Fragment-based drug design does benefit from the potential of developing customized compound–protein interactions, resulting in a greater capacity for optimization. By growing compounds from smaller fragments, it is also possible to explore a wider chemical space.

## Other Comparisons of Conventional and Structure-Based Drug Design High-Throughput Screening

Although not common, several other research groups have performed parallel conventional HTS and *in silico* screens of the same biological target. These have targeted host proteins such as the A_2A_ adenosine receptor (Chen et al., 2013), protein tyrosine phosphate-1B (Doman et al., 2002), angiogenin (Jenkins et al., 2003), and glycogen synthase kinase-3β (Polgar et al., 2005), proteins which are targets for the treatment of Parkinson's disease, type 2 diabetes, cancer, and Alzheimer's disease, respectively. Complementary screens to identify pathogen inhibitors have targeted cruzaine, an essential cysteine protease in *Trypanosoma cruzi* (causative agent of Chagas disease) (Ferreira et al., 2010), and dihydrodipicolinate reductase, an essential *Mycobacterium tuberculosis* protein (Paiva et al., 2001). However, neither of these studies ultimately tested the activity of identified hit compounds against the target pathogen. All of the above studies were successful in identifying active compounds through both their HTS and *in silico* approaches with similar properties. Perhaps not unexpectedly, however, the structures identified depended on the libraries used; when using the same library, both HTS and *in silico* screens identified similar classes of structures (Polgar et al., 2005). *In silico* HTS approaches generally were able to generate a list of compounds enriched in active hits by 20-fold or more compared with the traditional HTS approaches, but this often came at the cost of fewer active compounds identified. This shortcoming could be addressed by lowering the threshold for selecting compounds from the *in silico* screen, although this would also reduce the enrichment of true hits in the selected library. However, the ability of *in silico* screens to assess orders of magnitude more compounds than traditional HTS is also able to compensate for the lower overall hit rate.

Our experience as outlined here indicates that both SBDD and HTS are indeed able to identify novel compounds with comparable activities, and that SBDD is able to identify a small subset of compounds highly enriched in active molecules. The trade-off between the two approaches is the high speed with which the SBDD can screen an extensive library to produce a shortlist of compounds consistently enriched in active compounds compared with the ability of the traditional HTS screen to routinely identify more varied active compounds. Using the information from the crystal structure for a known ligand will greatly increase the likelihood of success in an *in silico* screen. It would seem, therefore, that for targets where a large number of active compounds are likely to exist, an *in silico* screening approach would be strongly recommended in order to expedite the early phase of hit identification while still identifying a sufficient number of hits. For targets with a known structure SBDD would be appropriate, while LBDD is well suited when a large number of active compounds are known. In contrast, where active compounds are likely to be rare, computational screening runs the risk of missing potentially valuable compounds, meaning that a larger, albeit more involved, HTS may be favored. Finally, tandem/parallel conventional HTS followed by *in silico* screening is a powerful approach, with *in silico* approaches being particularly valuable for selection of compounds/scaffolds for focused SAR around hits from conventional HTS.

## Conclusion

This focused review provides, to our knowledge, the first assessment of conventional HTS and *in silico* screening for inhibitors of a host–pathogen protein–protein interaction interface, with antivirals as the end product. Consistent with other studies that have performed screens in a comparable fashion, but for inhibitors of very different target proteins/enzymes with other outcomes in mind (see above), it is clear from this case study that combined and iterative HTS and *in silico* screens afford complementary strengths to the task of novel drug identification. Traditional HTS can always be enhanced by insightful computational modeling based on ever-advancing structural inputs that are able to incorporate more of the physicochemical properties of the compounds, existing information about active compounds, and known off-target effects to help identify a compound library enriched in hits for experimental assessment. In the same way, results from traditional HTS can be a great starting point for *in silico* approaches to identify related compounds/structures for testing and optimization. Importantly, as available computational power increases, the ability to extract new information from traditional screens will undoubtedly encourage the incorporation of contributions from *in silico* CADD more and more in the future.

## Author Contributions

SS and DT wrote the review article. DJ redacted the manuscript and figures into its final form. All authors approved the submitted version.

## Conflict of Interest

The authors declare that the research was conducted in the absence of any commercial or financial relationships that could be construed as a potential conflict of interest.
